# Aged *Pericarpium Citri Reticulatae ‘Chachi’* Attenuates Oxidative Damage Induced by *tert*-Butyl Hydroperoxide (*t*-BHP) in HepG2 Cells

**DOI:** 10.3390/foods11030273

**Published:** 2022-01-20

**Authors:** Qian Yu, Yexing Tao, Yuting Huang, Daniel Zogona, Ting Wu, Ruiting Liu, Siyi Pan, Xiaoyun Xu

**Affiliations:** 1 College of Food Science and Technology, Huazhong Agricultural University, Wuhan 430070, China; yuqian_68@webmail.hzau.edu.cn (Q.Y.); yexing.tao@webmail.hzau.edu.cn (Y.T.); huangyuting116024@webmail.hzau.edu.cn (Y.H.); zdanilo27@gmail.com (D.Z.); ting.wu@mail.hzau.edu.cn (T.W.); rating@webmail.hzau.edu.cn (R.L.); pansiyi@mail.hzau.edu.cn (S.P.); 2 Key Laboratory of Environment Correlative Dietology (Ministry of Education), Huazhong Agricultural University, Wuhan 430070, China

**Keywords:** *Pericarpium Citri Reticulatae ‘Chachi’*, flavonoids, polymethoxyflavones, HepG2 cell, oxidative damage

## Abstract

This study investigated the protective effects of aged *Pericarpium Citri Reticulatae ‘Chachi’* (PCR-C) on *tert*-butyl hydroperoxide (*t*-BHP)-induced oxidative damage in HepG2 cells. According to HPLC analysis, PCR-C aged 10 years (PCR-C10) had the highest flavonoids content, especially polymethoxyflavones (PMFs), compared with the fresh peel of *Citrus reticulata cv. ‘Chachiensis’* and PCR-C aged 1, 3, and 5 years. Then, flavonoids-rich PCR-C samples and non-flavonoids-rich PCR-C samples (NF) were prepared by extracting and purifying PCR-C of different aging periods, for further cell experiments. Pretreatment with flavonoids-rich PCR-C samples (particularly PCR-C10) considerably reversed *t*-BHP-induced oxidative damage in HepG2 cells by improving cell viability, increasing SOD activity and GSH levels and reducing the overproduction of ROS and MDA. Correlation analysis further indicated that the accumulation of PMFs, mainly 5,6,7,4′-tetramethoxyflavone and nobiletin, was the main reason that PCR-C10 maintained the redox balance in HepG2 cells. These findings provided direct evidence for the cellular antioxidant activity of aged PCR-C and a guide for PCR-C’s classification, authentication and rational use.

## 1. Introduction

*Pericarpium Citri Reticulatae* (PCR), called “Chenpi” in Chinese, is the dried ripe pericarp of *Citrus reticulata Blanco* or its cultivars. PCR is not only widely consumed as a dietary condiment, tea ingredients, and various snacks [1], and also used as a traditional medicine to treat indigestion and respiratory diseases [2]. *Pericarpium Citri Reticulatae ‘Chachi’* (PCR-C), known as “Guangchenpi” in Chinese, is considered as a premium PCR in Chinese and the United States Pharmacopoeia, and is very popular among consumers in the international market due to its remarkable medicinal and health benefits [3]. According to ancient Chinese books, PCR-C is “the older, the better”, which is still used today [4]. However, the effect of aging on the quality and efficacy of PCR-C has not been clearly described so far.

Excessive reactive oxygen species (ROS) production is a major factor in the induction and development of many oxidative stress-related diseases such as inflammation and cardiovascular diseases [5]. A previous study reported that dieted rich in natural plant polyphenols could effectively reduce and prevent the occurrence of various diseases related to oxidative stress damage [6]. Moreover, PCR-C is a potential antioxidant rich in flavonoids such as hesperidin, nobiletin, and tangeretin [3,7,8]. In the past, the antioxidant activity of flavonoids in citrus peel or PCR-C was mainly evaluated by chemical analysis methods such as ABTS, DPPH, and ORAC [9,10,11]. However, the above methods had limitations: they broke away from the environment conditions in the organism and ignored the bioavailability of flavonoids, resulting in a large discrepancy between the measured results and the actual results [12].

Compared with chemical methods, the cell oxidative damage model can better reflect the antioxidant response in organisms under oxidative damage. Human hepatocarcinoma HepG2 cell lines is a well-differentiated transformed cell line, which is easy to culture and well characterized [13]. Therefore, the oxidative damage model established by HepG2 cells has been widely used in cell-based bioassays of food antioxidant activity [14,15,16]. *Tert*-butyl hydroperoxide (*t*-BHP), an ideal inducer of oxidative damage at present, can react with transition metals in cells to produce free radicals, which can initiate ROS formation, thereby inducing oxidative damage in cells [13]. Goya et al. [17] also confirmed the t-BHP-induced HepG2 cell oxidative damage model, which can be used as a model for the study of different antioxidant mechanisms of dietary compounds. However, studies on the protective effect of PCR-C on *t*-BHP-induced oxidative damage in HepG2 cells have not been reported. Therefore, this study aimed to investigate the protective effect of PCR-C of different aging periods on *t*-BHP-induced oxidative damage in HepG2 cells. In addition, this study sought to reveal the effect of prolonged aging time on the flavonoids in PCR-C and explored the correlation between changes in bioactive components and the cellular antioxidant activity in PCR-C.

## 2. Material and Methods

### 2.1. Samples

PCR-C aged for 1, 3, 5, 10 years and the fresh peel of *Citrus reticulata cv. ‘Chachiensis’* (Fresh) were purchased from Ganze Garden, Shuangshui Town, Xinhui District, Jiangmen City, Guangdong Province and authenticated by the Pharmacy Faculty, Hubei university of Chinese Medicine. The fresh *Citrus reticulata cv. ‘Chachiensis*’ was picked in December 2020, which is the mature period of *Citrus reticulata cv. ‘Chachiensis*’. After harvest, they were washed with clean water, artificially peeled, and naturally air-dried at room temperature (25–30 °C). It is noteworthy that the samples of different aging periods were from different batches of *Pericarpium Citri Reticulatae ‘Chachi’*, but their varieties, origins, and aging methods were consistent. In this study, the influences of uncontrollable factors such as sunshine duration, rainfall, temperature, atmospheric or soil conditions on the samples were considered to be consistent.

### 2.2. Chemicals and Reagents

The reference standards of 5,6,7,4′-tetramethoxyflavone, 5,7,8,4′-tetramethoxyflavone, hesperidin, nobiletin, tangeretin, sinensetin, isosinensetin, 5-demethylnobiletin, 3,5,6,7,8,3′,4′-heptamethoxyflavone were purchased from Shanghai yuanye Bio-Technology Co., Ltd. (Shanghai, China). Gallic acid was also purchased from the same company. Rutin, 2′,7′-dichlorofluorescein diacetate (DCFH-DA) and *t*-BHP were obtained from Sigma Chemical Co. (St. Louis, MO, USA). The following agents were in HPLC grade: acetonitrile and methanol were purchased from Thermo Fisher Scientifc, Inc. (Waltham, MA, USA), formic acid purchased from Tianjin Kemiou Chemical Reagent Co., Ltd. (Tianjin, China). Deionized Milli-Q water (18 MΩ·cm) (Millipore, Burlington, MA, USA) was used to prepare the mobile chromatographic phases. In addition, NaOH, HCl, ethanol, ethyl acetate and other solvents used for extraction separation and/or detection, which were of analytical grade, were obtained from Sinopharm Chemical Reagent Co., Ltd. (Shanghai, China). Minimum essential medium (MEM) was obtained from Gibco Laboratories (Life Technologies Inc., Grand Island, NY, USA). Fetal bovine serum (FBS) was obtained from AusGeneX (Brisbane, Qld, Australia). The 3-(4, 5-dimethylthiazol-2-yl)-2, 5-diphenyltetrazolium bromide (MTT) were obtained from Gen-View Scientific Inc. (Calimesa, CA, USA). Bicinchoninic acid (BCA) protein assay kit was purchased from Dingguo changsheng Technology Co., Ltd. (Beijing, China). Total Superoxide Dismutase (T-SOD) assay kit, Reduced glutathione (GSH) assay kit and Cell Malondialdehyde (MDA) assay kit were obtained from Nanjing Jiancheng Bioengineering Institute (Nanjing, China).

### 2.3. Sample Preparation

PCR-C of different storage periods and the fresh peel were dried to constant weight in a constant temperature air drying oven (WGL-45B, Taisite Ultrasonic Instrument Co., Ltd., Tianjin, China) at 50 °C, smashed with a high-speed multifunctional pulverizer (YB-1000A, Yongkang Sufeng industry and Trade Co., Ltd., Jinhua, China), and screened with 60 mesh. The powder was stored at −20 °C until use.

### 2.4. Extract Preparation of Free and Bound Phenolics

The dried samples (500 mg) were extracted twice in ethanol/water (80:20, *v*/*v*) with ultrasound assistance and subsequently concentrated to obtain the extractable (Free) fraction through evaporation. A proper volume of the obtained solution was removed and filtered through a 0.45 μm of filter membrane for HPLC analysis. The remaining solution was used for the determination of total phenolics and flavonoids. The extraction of bound phenolics referred to the reported method [18] with moderate modification. The residue from the free phenolic extraction was mixed with 2.5 mL 4 mol/L NaOH for 1 h with shaking under nitrogen gas at room temperature. After adjusting the pH to 2 ± 0.2 with 6 mol/L HCl, the mixture was extracted five times with ethyl acetate. The pooled ethyl acetate fraction was evaporated and then reconstituted with methanol for a bound phenolic fraction. The solution was filtered using the same procedure and stored at −80 °C for HPLC analysis, while the remaining reconstituted solution was stored at −20 °C.

High performance liquid chromatography–quadrupole-time-of-flight tandem mass spectrometry (HPLC-Q-TOF-MS/MS) analysis solution needs to be purified on the SPE Vac 3cc C18 column (Waters, Milford, MA, USA). Then, the elution was freeze-dried in a vacuum to obtain a powder. Finally, the powder was reconstituted with methanol. The reconstituted solution was filtered through a 0.22 μm filter membrane before analysis.

### 2.5. Determination of Total Phenolics and Total Flavonoids Concentration

The total phenolics concentration (TPC) in both the Free and Bound fractions (Free-TPC and Bound-TPC, respectively) was measured using Folin-Ciocalteu reagent with gallic acid as a standard as described in Choi et al. [4]. The total flavonoids concentration (TFC) in both the Free and Bound fractions (Free-TFC and Bound-TFC, respectively) was measured using the protocol in Wang et al. [19]. TPC and TFC were expressed as mg of gallic acid equivalents (GAE)/g dry weight (dw) and mg of rutin equivalents (RE)/g dry weight (dw), respectively.

### 2.6. Determination of Free and Bound Flavonoids in PCR-C

PCR-C samples analysis was carried out using an HPLC system equipped with a binary pump, an online degasser, an autosampler, and a photodiode-array detector (PDA) (Agilent Technologies Inc., Santa Clara, CA, USA). Chromatographic separation of the free and bound extracts was performed by using an Agilent ZORBAX SB-C18 analytical column (250 × 4.6 mm i.d., 5 μm; Agilent Technologies Inc., Santa Clara, CA, USA). The column was placed in a column oven set at 25 °C. The injection volume was 5 μL, and the mobile phase was composed of 0.1% aqueous formic acid (*v*/*v*) (phase A) and acetonitrile (phase B) at a constant flow rate of 1 mL/min. The gradient was as follows: 15–25%B (0–5 min), 25–35%B (5–20 min), 35–45%B (20–40 min), 45–75%B (40–45 min), 75–85%B (45–50 min), 85–15%B (50–55 min), and constant 15% B for 5 min. The effluent was monitored by UV detection at 280 and 330 nm.

MS/MS identification was conducted with a quadrupole time-of-flight mass spectrometer (6520B Q-TOF; Agilent Technologies Inc., Santa Clara, CA, USA) equipped with an electrospray ionization (ESI) source. Using a shunt device, the flow rate after entering the mass spectrometry detection was changed to 0.5 mL/min. The electrospray ionization source operated in both positive and negative ionization modes. The scanning range of MS acquisition mode is from *m*/*z* 60 to 1500. Subsequent activation of the three most intense precursor ions by MS/MS using a collision energy of 10, 30, and 50 eV. The results were analyzed by Q-TOF MASSHUNTER WORKSTATION™ software (version B.04.00SP3 Profinder, Agilent Technologies, Santa Clara, CA, USA).

### 2.7. Purification of Free Flavonoids in PCR-C

Followed the method in Section 2.4 to extract free flavonoids from samples, and then the crude extract (500 mg/mL) was added to a chromatography column (5 cm × 50 cm) equipped with HPD300 resin, as previously described [20], with moderate modification. The eluent was eluted with eight bed volume (BV) distilled water, then collected the eluent to obtain the non-flavonoids fraction 1. Then eluent was eluted with 6BV ethanol/water (7:93, *v*/*v*), and the eluent was collected to obtain the non-flavonoid fraction 2. Finally, eluent was eluted with 6BV ethanol/water (80:20, *v*/*v*) to obtain the flavonoids components, eluted until the absorbance value of the eluent at 280 and 330 nm remained unchanged. All the eluents were evaporated and concentrated, then freeze-dried and stored at −80 °C. Before the cell experiment, the non-flavonoid fractions 1 and 2 of each sample were combined into non-flavonoid fraction, that is non-flavonoids-rich PCR-C samples (NF). The obtained samples were analyzed by HPLC again.

### 2.8. Cell Culture

Human hepatocellular carcinoma HepG2 cells were purchased from Stem Cell Bank, Chinese Academy of Sciences in Shanghai. HepG2 cells were cultured in MEM supplemented with 10% FBS, penicillin (100 U/mL) and streptomycin (100 μg/mL) in an atmosphere of 5% CO_2_ and 90% relative humidity at 37 °C.

### 2.9. MTT Assay

The cytotoxicity of NF and PCR-C samples was evaluated by MTT assay. The cells were grown in 96-well plates at a density of 2 × 10^4^ cells/well. After attachment, HepG2 cells were then incubated with NF and PCR-C samples (50 to 100 µg/mL) for 24 h or *t*-BHP (0–700 µM) for 3 h. The cells were further incubated with an MTT solution (0.5 mg/mL) for 4 h. Then, MTT solution was removed and DMSO (180 μL/well) was added to dissolve crystallized MTT. A microplate reader (MultiScan Go, Thermo Scientific Co., Ltd., Waltham, MA, USA) was used for measuring absorbance at 490 nm after shaking for 5 min. Five technical repeats were completed for NF and PCR-C.

### 2.10. Cytoprotective Effects

HepG2 cells (2 × 10^4^ cells/well) were cultured in 96-well plates. The cells were then pretreated with 50 µg/mL NF and PCR-C samples for 24 h, followed by challenging with *t*-BHP (300 µM) to induce oxidative stress. After 3 h incubation, the cell viability was determined.

### 2.11. Measurement of Intracellular ROS Levels

The intracellular ROS levels were quantified using the DCFH-DA probe, as previously described [21], with moderate modification. HepG2 cells (4 × 10^5^ cells/well) were plated in 24-well plates. After incubation with 50 μg/mL NF and PCR-C samples for 24 h, the cells were further incubated with DCFH-DA probes (10 µM) for 30 min. Then, the cells were washed three times with PBS. After removing the probe, 0.5 mL of serum-free medium with *t*-BHP (300 µM) or without was added per well, incubated for 1.5 h. The fluorescence intensity was measured using a Synergy HTX Multi-Reader (Bio-Tex, Winooski, VT, USA) (excitation, 485 nm; emission, 530 nm). Intracellular ROS levels were expressed as the fold to control group.

### 2.12. Assessment of MDA, T-SOD and GSH

To measure the MDA, T-SOD activity and GSH contents in HepG2 cells, the cells (1 × 10^6^ cells/well) were cultured in six-well plates. After attachment, cells were pretreated with 50 μg/mL NF and PCR-C samples for another 24 h and then incubated with *t*-BHP (300 µM, 3 h). After washing the cells three times with pre-cooling PBS, the cells were harvested and lysed in an ultrasonic bath. The MDA, T-SOD activity, and GSH contents were determined using the cell malondialdehyde (MDA) assay kit, total superoxide dismutase (T-SOD) assay kit and reduced glutathione (GSH) assay kit, respectively. The results were normalized to protein concentrations.

### 2.13. Data Analysis and Statistics

Statistical analyses were performed using SPSS for Windows version 25.0 (SPSS Inc., Chicago, IL, USA) and presented as the mean ± SD. The statistical significance of the data was determined using one-way analysis of variance (ANOVA). Duncan’s test and Tamhane’s test was used to compare the mean values among samples (*p* < 0.05). Correlation coefficient between four indicators related to cell antioxidant activity (ROS, MDA, T-SOD, GSH) and contents of total flavonoids, PMFs, and nine main flavonoids after purification were done by Pearson’s correlation coefficient option.

## 3. Results and Discussion

### 3.1. Measurement of Total Phenolics and Flavonoids Contents in PCR-C during Aging

PCR-C contained flavonoids in free and bound forms. The bound fractions were combined with proteins, monosaccharides, organic acids through ester, glycoside, and ether glycoside links [18]. Investigating the changes in total phenolics and flavonoids contents of free and bound fractions in PCR-C was the first step to understand the material basis of PCR-C in the aging process.

**Figure 1:**
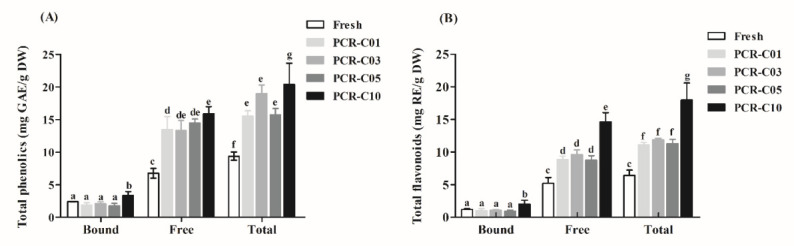
Free, bound, and total fraction concentrations in (**A**) total phenolics and (**B**) total flavonoids of PCR-C samples at different storage periods. Data were expressed as the mean ± SD (*n* = 3). Mean values with different letters in the figure indicate significant differences among PCR-C of different storage periods (*p* < 0.05).

Phenolics (Figure 1A) and flavonoids (Figure 1B) concentrations remarkably increased in free and bound fractions during aging. In line with our results, Wang et al. [3] found a dramatic accumulation of bound phenolics and flavonoid contents during aging, when they compared phenolics and flavonoid contents in fresh and dried *Citrus reticulata cv. ‘Chachiensis*’ pericarp samples, as well as PCR-C aged 5- and 12-years samples. However, the Free-TPC had no significant variations in their study. In contrast, another study found that Free-TPC in PCR increased significantly during storage, while Bound-TPC were relatively constant [4]. The differences between our results and those of previous studies might be due to the differences in the cultivars and aging periods of the samples. Zheng et al. [22] compared the similarity and difference among various cultivars through principal component analysis (PCA). The results showed that compared with *Citrus reticulata cv. ‘Chachiensis’*, the content of flavonoids in *Citrus. reticulata ‘Tankan’* was lower, while the content of flavonoid in *Citrus. reticulata ‘Suavissima’* was higher. Luo et al. [23] found that compared with PCR stored 3–5 years, the flavonoid contents in PCR stored 10 years were significantly higher.

### 3.2. Tracing the Variation of Free and Bound Flavonoids in PCR-C during Aging

The results in Section 3.1 showed that the TFC increases continuously during aging. In this section, the changes in composition and contents of the individual flavonoids from the free and bound fractions in PCR-C during aging were investigated.

Free and bound flavonoids in PCR-C samples were analyzed by HPLC-Q-TOF-MS/MS in positive and negative ion modes. This was the first mass spectrometric identification of bound flavonoids in PCR-C. The elution time, accurate molecular weights, and MS/MS fragment ions of identified flavonoids were listed in Table 1 and Table 2.

A total of 17 major constituents were identified in PCR-C free flavonoids, and tentatively characterized (Table 1), and 20 major constituents were identified in PCR-C bound flavonoids, and tentatively characterized (Table 2). However, the chemical structures of compounds 2 and 6 (Table 2) needed to be further confirmed. The total ion current (TIC) chromatograms corresponding to positive and negative signals of free and bound flavonoids in PCR-C were shown in Supplementary Figure S1. According to the response intensity, nine flavonoids (hesperidin, isosinensetin, sinensetin, 5,7,8,4′-tetramethoxyflavone, nobiletin, 5,6,7,4′-tetramethoxyflavone, 3,5,6,7,8,3′,4′-heptamethoxyflavone, tangeretin, 5-demethylnobiletin) with high response intensity were selected for HPLC quantitative analysis. The linear regression equation, linear range, correlation coefficient, the limit of detection (LOD), the limit of quantification (LOQ), intra- and inter-precision, repeatability, and recovery of the nine flavonoids were shown in Supplementary Table S1. A chromatogram of the reference standards was displayed in Supplementary Figure S2. At the two detection wavelengths (280 and 330 nm), the chromatographic peaks of the nine flavonoids presented a good shape and the baselines were stable.

**Figure 2:**
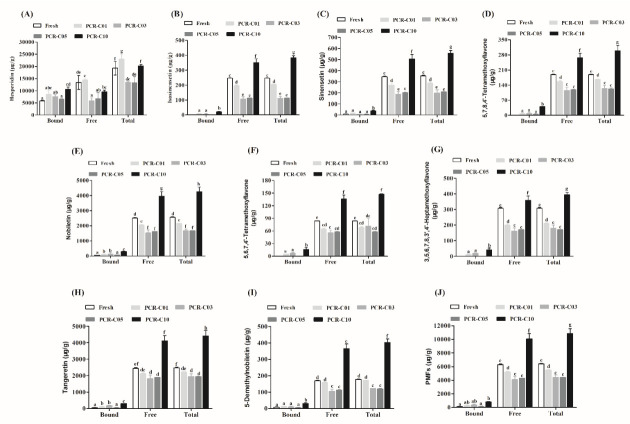
The variation of nine free, bound, and total flavonoids in PCR-C samples at different storage periods. (**A**) hesperidin, (**B**) isosinensetin, (**C**) sinensetin, (**D**) 5,7,8,4′-tetramethoxyflavone, (**E**) nobiletin, (**F**) 5,6,7,4′-tetramethoxyflavone, (**G**) 3,5,6,7,8,3′,4′-heptamethoxyflavone, (**H**) tangeretin, (**I**) 5-demethylnobiletin, (**J**) PMFs. Data were expressed as the mean ± SD (*n* = 3). Mean values with different letters in the figure indicate significant differences among PCR-C of different storage periods (*p* < 0.05).

The variation of nine free, bound, and total flavonoids in PCR-C during aging was shown in Figure 2. Among the main free flavonoids in the Fresh and PCR-C of different aging periods, free hesperidin was the highest content, accounting for 68.11% (Fresh), 73.63% (PCR-C01), 58.97% (PCR-C03), 61.30% (PCR-C05), and 48.72% (PCR-C10) of the total free flavonoids, respectively. Consistent with the present study, the content of hesperidin in sixteen PCR samples was the highest, ranging from 27.15 to 86.90 mg/g [24], followed by nobiletin and tangeretin, and the content of 5,6,7,4′-tetramethoxyflavone was the lowest. The content of eight free PMFs (isosinensetin, sinensetin, 5,7,8,4′-tetramethoxyflavone, nobiletin, 5,6,7,4′-tetramethoxyflavone, 3,5,6,7,8,3′,4′-heptamethoxyflavone, tangeretin, 5-demethylnobiletin) in PCR-C10 was significantly higher than in the other samples (*p* < 0.05, the exact values of *p* were shown in Supplementary Table S2). The bound hesperidin was the most abundant bound flavonoids in the Fresh and PCR-C of different aging periods, accounting for about 85–98% of the total. Bound isosinensetin, 5,6,7,4′-tetramethoxyflavone, 3,5,6,7,8,3′,4′-heptamethoxyflavone were not detected in PCR-C05 and Fresh. Besides, bound 5,7,8,4′-tetramethoxyflavone was not detected in Fresh either. However, after 10 years of aging, the contents of bound flavonoids in PCR-C were significantly higher than those in the other samples (*p* < 0.05). Obviously, the total contents of eight PMFs (isosinensetin, sinensetin, 5,7,8,4′-tetramethoxyflavone, nobiletin, 5,6,7,4′-tetramethoxyflavone, 3,5,6,7,8,3′,4′-heptamethoxyflavone, tangeretin, 5-demethylnobiletin) in PCR-C10 were the highest among the five samples (*p* < 0.05). Our results are different from the findings of Luo et al. [23] who reported that the contents of 5-dimethylnobiletin and naringin in PCR-C increased first and then decreased. The reasons of the differences could be related to the different origins [25], cultivars [22,26], PCR-C harvest period [27], and storage conditions [28]. However, using PCA and orthogonal partial least squares discriminant analysis, Luo et al. [23] found that samples aged for 3, 5, and 10 years were clearly separated, indicating that the metabolites changed significantly during aging. This finding was consistent with our study.

We found that the contents of free and bound flavonoids in Figure 2 were significantly lower after 3 and 5 years of aging, which were consistent with the results in 3.1. This might be because that the dominant bacterial community on the surface of PCR-C had changed during aging [29]. Meanwhile, the contents of PMFs and hesperidin showed an increasing trend during the aging process, which may be related to two aspects of compounds transformation. One is the transformation between free and bound flavonoids [4]. However, the contents of free and bound flavonoids showed an increasing trend and did not maintain a dynamic balance during aging, which was consistent with the results in Section 3.1. Therefore, we presumed that the transformation between free and bound flavonoids was the secondary factor affecting the changes. The other one is the transformation of other phenolics to flavonoids. We consider this aspect as the main factor for the gradual increase in flavonoids content. At present, relevant studies have pointed out that through microbial transformation, the content of medicinal substances in Chinese herbal medicines could be increased or new active ingredients could be obtained, leading to the improvement of the original Chinese herbal medicines [30,31]. Additionally, He et al. [29] found that the two main dominant genera in PCR-C were *Bacillus* and *Lactococcus*, and the increase of the content of nobiletin was related to the existence of those genera. Therefore, during the aging process, the transformation of other phenolics into flavonoids was achieved through the microorganism metabolism. Furthermore, flavonoids are widely recognized as the main active ingredients in PCR-C, and the increase of their content is of particular importance for improving the quality of PCR-C.

### 3.3. Purification of Free Flavonoids in PCR-C

**Figure 3:**
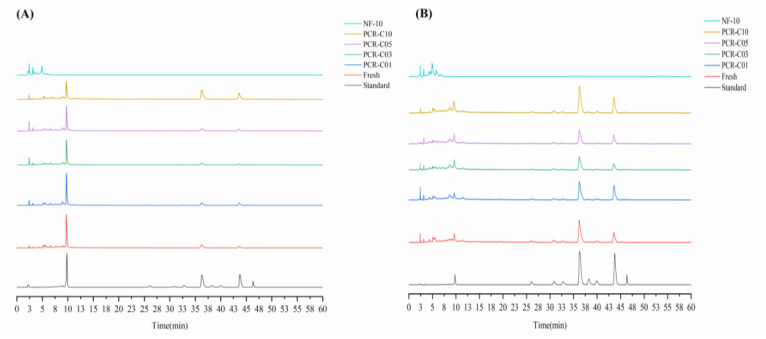
Chromatogram of five PCR-C samples, non-flavonoids-rich PCR-C10 (NF-10), and standard at 280 (**A**) and 330 nm (**B**).

Currently, most studies on the antioxidant activity of PCR-C have been carried out with PCR-C extracts or the common flavonoids as the experimental subjects [9,11,32]. In this study, for the first time, flavonoids-rich PCR-C samples and non-flavonoids-rich PCR-C samples (NF) were extracted and purified from PCR-C of different aging periods. The flavonoids-rich PCR-C samples and non-flavonoids-rich PCR-C10 (NF-10) were used as raw materials for cells experiment. This could more accurately determine the main chemical components in PCR-C that exert antioxidant activity. The total flavonoids contents of PCR-C samples before and after purification was determined, and the results were presented in Table 3. After treatment with HPD300 resin, the total flavonoids contents in the samples increased by more than 10 folds, indicating that HPD300 resin could effectively adsorb flavonoids to achieve the purpose of purification. Consistent with the present study, Tang et al. [33] also found that the total flavonoids content increase by 4.55 folds after using HPD300 resin to adsorb flavonoids from *Fortunella margarita* peel. After purification, the change trend of total flavonoids content was consistent with the results in the Section 3.1. Moreover, the content of total flavonoids in the purified PCR-C10 was still significantly higher than in other samples (*p* < 0.05). The six obtained samples (NF-10, Fresh, PCR-C01, PCR-C03, PCR-C05, PCR-C10) after purification were analyzed by HPLC, and the results were presented in Figure 3. Comparing the HPLC chromatograms of NF and PCR-C samples, we observed that the nine main flavonoids were not present in NF, which indicated that those flavonoids in PCR-C were completely retained after adsorption by the HPD300 resin. Pigments, soluble sugars, and soluble proteins were eluted with distilled water and ethanol/water (7:93, *v*/*v*). After treatment with HPD300 resin, the contents of nine flavonoids in PCR-C samples were displayed in Table 4. After purification, the contents of hesperidin, tangeretin and 5-demethylnobiletin showed a fluctuating trend. The content changes of sinensetin, nobiletin, 5,6,7,4′-tetramethoxyflavone, 3,5,6,7,8,3′,4′-heptamethoxyflavone, and PMFs were consistent with the corresponding free flavonoids in Section 3.2. Hesperidin (50.2–67.6%), nobiletin (15.2–24.7%), and tangeretin (9.1–17.8%) considerably contributed to the total flavonoids content in the purified PCR-C samples. Compared with the results in the Section 3.2, it was found that after purification, the contents of the nine flavonoids were increased by about 5–20 folds.

### 3.4. Cell Viability

**Figure 4:**
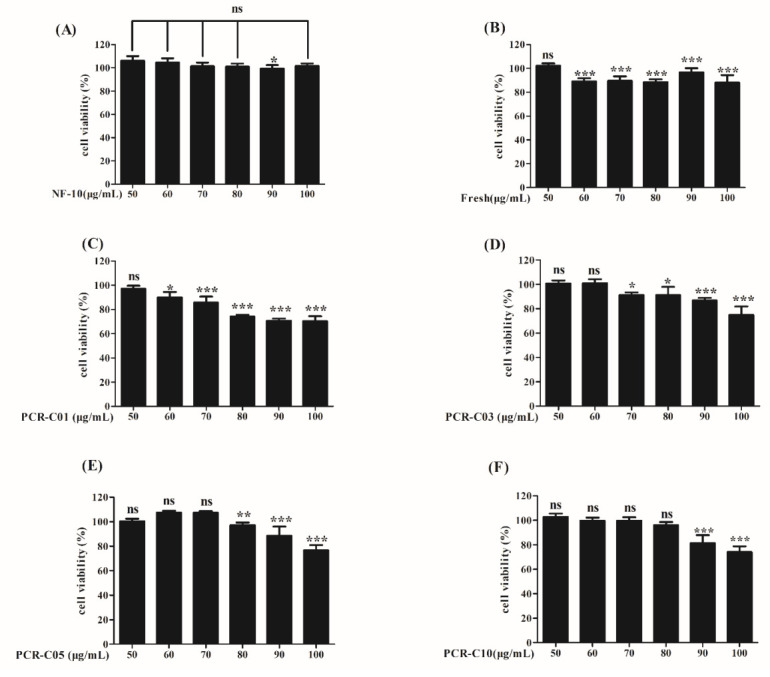
The cytotoxicity of NF-10 (**A**), Fresh (**B**), PCR-C01 (**C**), PCR-C03 (**D**), PCR-C05 (**E**), PCR-C10 (**F**) in HepG2 cells. HepG2 cells were treated with various concentrations of samples (50–100 μg/mL) for 24 h, and then cell viability was analyzed by MTT assays. Results are presented as mean ± SD, *n* = 6. Statistical comparisons were made using the Student’s *t*-test. ns means no significant difference. * *p* < 0.05, ** *p* < 0.01, *** *p* < 0.001 vs. control group.

To ensure that the purified samples used in subsequent experiments were not toxic to cells, it was necessary to determine the non-toxic concentration range of the purified samples. The cytotoxicity of NF-10, Fresh, PCR-C01, PCR-C03, PCR-C05 and PCR-C10 on HepG2 cells was evaluated by the MTT method. HepG2 cells were incubated with six of the purified samples (50 to 100 μg/mL) for 24 h. Within the concentration range 50 to 80 μg/mL, NF-10 and PCR-C10 did not exhibit cytotoxic effects compared with control groups (Figure 4A,F). Fresh and PCR-C01 at 50 μg/mL did not exhibit cytotoxic effects compared with control groups (Figure 4B,C). PCR-C03 at 50 and 60 μg/mL did not exhibit cytotoxic effects compared with control groups (Figure 4D). Within the concentration range of 50 to 70 μg/mL, PCR-C05 did not exhibit cytotoxic effects compared with control groups (Figure 4E). Thus, 50 μg/mL was selected as the non-toxic concentration of samples for further experiments in this study.

### 3.5. Purified PCR-C Samples Attenuated t-BHP-Induced Cytotoxicity

**Figure 5:**
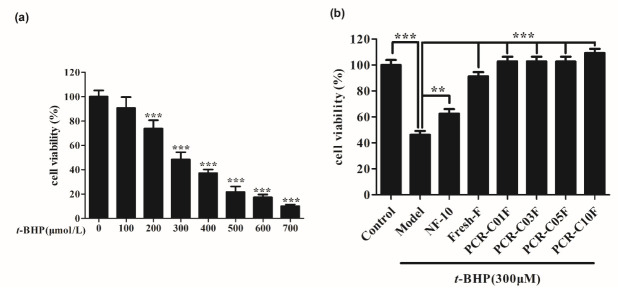
Effect of *t*-BHP on cell viability and protective effects of PCR-C of different storage periods, Fresh and NF on *t*-BHP-induced cytotoxicity. (**a**) HepG2 cells were treated with *t*-BHP (0–700 µM) for 3h and cell viability was analyzed by MTT assay. (**b**) HepG2 Cells were pretreated with 50 µg/mL NF-10, Fresh, PCR-C01, PCR-C03, PCR-C05, and PCR-C10 for 24h prior to exposure to *t*-BHP (300 µM) for 3 h, then cell viability was determined by MTT assay. Results are presented as mean ± SD, *n* = 6. ** *p* < 0.01, *** *p* < 0.001 vs. control group.

*t*-BHP could not only react with biological macromolecules on cell membrane to cause oxidative damage, but also diffuse into the cell membrane and react with transition metals in cells to produce cytotoxic free radicals, further causing cell damage [13]. In this study, after incubating HepG2 cells with *t*-BHP (0 to 700 µM) for 3 h, the cell viability was notably decreased to 10% (*p* < 0.001), which indicated that *t*-BHP-induced HepG2 cell oxidative damage model was successfully constructed. The results revealed that IC50 value was 301.10 ± 19.70 µM (Figure 5a). Accordingly, a concentration of 300 µM *t*-BHP was used for the subsequent experiment.

The cytoprotective effect of NF-10 and purified PCR-C samples against *t*-BHP (300 µM, 3 h) induced-cytotoxicity was shown in Figure 5b. Results indicated that five purified PCR-C samples markedly attenuated *t*-BHP-induced cytotoxicity (*p* < 0.001). And their cytoprotective effect on HepG2 cells was significantly stronger than that of NF-10 (*p* < 0.001). Our results were consistent with the findings of Jannat et al. [34] who demonstrated that sweet orange (*Citrus sinensis* (L.) *Osbeck*) juice powder rich in hesperidin could improve the decline of HepG2 cell viability caused by *t*-BHP treatment. In addition, Hwang and Yen [35] reported that hesperidin could significantly inhibit the reduction of PC12 cell viability induced by H_2_O_2_. A previous study in our laboratory also found that tangeretin could markedly attenuate *t*-BHP-induced cytotoxicity in a dose-dependent manner (*p* < 0.01) [36]. In this study, the flavonoids might be responsible for the cytoprotective effect of purified PCR-C.

### 3.6. Purified PCR-C Samples Attenuated t-BHP-Induced Oxidative Damage

**Figure 6:**
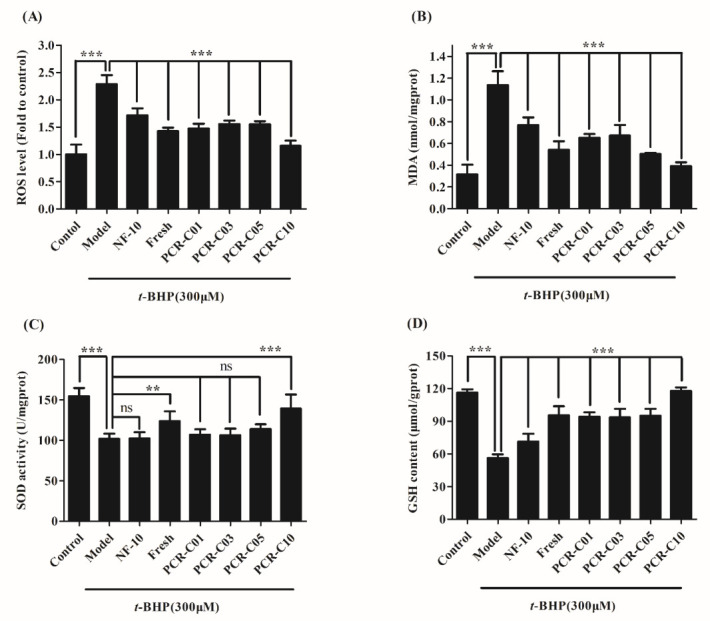
Effect of PCR-C of different storage periods, Fresh and NF-10 on *t*-BHP-induced ROS (**A**), and MDA (**B**) generation and reduced SOD (**C**) and GSH (**D**) depletion. (**A**) HepG2 cells were pretreated with 50 µg/mL NF-10, Fresh, PCR-C01, PCR-C03, PCR-C05, and PCR-C10 for 24 h, then incubated with DCFH-DA for 30 min at 37 °C, then with *t*-BHP (300 µM) for 1.5 h. ROS levels were determined by a microplate fluorimeter. (**B**–**D**) HepG2 cells were pretreated with 50 µg/mL NF-10, Fresh, PCR-C01, PCR-C03, PCR-C05, and PCR-C10 for 24h, and then incubated with *t*-BHP (300 µM) for 3 h. MDA, SOD activity, and GSH content were determined using MDA, T-SOD and GSH assay kit, respectively. Results are presented as mean ± SD, *n* = 6, ns means no significant difference. ** *p* < 0.01, *** *p*< 0.001 vs. control group.

The effects of NF-10 and PCR-C of different aging periods on *t*-BHP-induced ROS generation was shown in Figure 6A. In this study, ROS levels in HepG2 cells increased significantly after treatment with *t*-BHP for 1.5 h (*p* < 0.001), which was about 2.3 folds higher than ROS levels in the control group. However, pretreatment of HepG2 cells with 50 µg/mL NF-10, Fresh, and PCR-C of different aging periods significantly inhibited the increase of intracellular ROS (*p* < 0.001). The effect was more prominent with 50 µg/mL purified PCR-C10, which brought ROS to a level similar to that of the control group. The above results demonstrated that NF-10, Fresh and PCR-C of different aging periods (in particular purified PCR-C10) could inhibit *t*-BHP-induced ROS generation, restore the cellular redox balance, and play the role of antioxidant to protect HepG2 cells. It was reported that PMFs such as nobiletin, tangeretin, sinensetin, and tetramethoxyflavones, were the most characteristic markers that distinguish the PCR-C from other PCR [37]. Nobiletin and tangeretin have been shown to reduce the overproduction of ROS in cells, respectively [38,39]. Therefore, it could be speculated that the ROS inhibition by aged PCR-C in HepG2 cells may be related to PMFs.

As a biomarker of lipid peroxidation, MDA could indirectly reflect the degree of cell damage [40,41]. The effects of NF-10 and PCR-C of different aging periods on *t*-BHP-induced MDA generation was shown in Figure 6B. In this study, *t*-BHP significantly increased MDA level in HepG2 (1.14 ± 0.13 nmol/mgprot) compared to the control group (0.32 ± 0.09 nmol/mgprot) (*p* < 0.001). However, pretreatment of HepG2 cells with 50 µg/mL NF-10, Fresh and PCR-C of different aging periods remarkably decreased the intracellular MDA level (*p* < 0.001 vs. model group) with purified PCR-C10 having the better effect. Indeed, we noticed that purified PCR-C10 exhibited similar MDA level with that of the control group (0.39 ± 0.03 nmol/mg prot and 0.32 ± 0.09 nmol/mg prot, respectively). The above results demonstrated that the NF-10, Fresh, and PCR-C of different aging periods could inhibit the increase of MDA level (with the purified PCR-C10 having the highest inhibitory effect), improve the degree of intracellular lipid peroxidation, and HepG2 cell damage, thus exerting an antioxidant effect.

The effects of PCR-C of different aging periods, Fresh and NF-10 on *t*-BHP-induced SOD depletion was shown in Figure 6C. SOD activity was notably lower by 34.1% in HepG2 cells treated with 300 µM *t*-BHP alone (model group) compared to the control group (*p* < 0.001). No difference in SOD activity was observed among Model, NF-10, PCR-C01, PCR-C03, and PCR-C05 groups (Figure 6C). Indicating that purified NF-10, PCR-C01, PCR-C03, and PCR-C05 did not reverse *t*-BHP-induced SOD depletion. Conversely, purified Fresh and PCR-C10 effectively restored SOD depletion induced by *t*-BHP in HepG2 cells (*p* < 0.01 and *p* < 0.001, respectively, Figure 6C). It was noticed that HepG2 cells pretreated with purified PCR-C10 had similar SOD activity with that of the control group. SOD was reported as a major superoxide scavenger by transforming superoxide radicals to H_2_O_2_, is subsequently converted to H_2_O by catalase (CAT) and glutathione peroxidase (GSH-Px) [42]. Therefore, SOD protected cells from attack by superoxide radicals [43]. Simultaneous determination of SOD activity and MDA level, could reflect the ability to scavenge oxygen free radicals, and the severity of the cells being attacked by oxygen free radicals [44]. Our results showed that purified PCR-C10 could inhibit *t*-BHP-induced intracellular MDA generation, and enhance SOD activity, thereby exerting greater antioxidant effect.

GSH is an essential endogenous small-molecule antioxidant which could restore and maintain the redox balance in cells by directly eliminating ROS, thereby inhibiting oxidative stress damage [45,46]. The effects of PCR-C of different aging periods, Fresh, and NF-10 on GSH content in HepG2 cells exposed to *t*-BHP was shown in Figure 6D. In this study, GSH content was significantly reduced by 51.7% in HepG2 cells treated with 300 µM *t*-BHP alone compared to the control group (*p* < 0.001). However, pretreatment of HepG2 cells with 50 µg/mL NF-10, Fresh, and PCR-C of different aging periods significantly repressed *t*-BHP-induced intracellular GSH reduction (*p* < 0.001). The inhibitory effect was more prominent with purified PCR-C10 pretreatment, which effectively reversed the action of *t*-BHP, leading to GSH content equivalent to that of control group. The results indicated that purified PCR-C10 could positively regulate the content of GSH, thereby protecting cells from oxidative damage. Consistent with the present study, Zong-Tsi et al. [47] also found that water extracts of sweet orange (*Citrus sinensis*) peel (WESP) dose-dependently increased GSH content in HepG2 cells, which was related to hesperidin, hesperetin, nobiletin, and tangeretin in WESP. Hence, it could be speculated that most likely purified PCR-C samples positively regulated GSH level due to their flavonoids content.

In some cases, the body antioxidant defense is not enough to control the excessive ROS production. That is why natural compounds with high antioxidant activity are important to help the body defense system [48]. Endogenous antioxidants such as SOD, CAT and GSH acted as the first line of defense against oxidative stress and were the important part in the scavenging system of ROS [43,46]. Wojdylo et al. [49] found that flavonoids and phenolic compounds could markedly retard oxidative degradation of lipids and improved the activity of cellular enzyme. PMFs such as nobiletin [50] and tangeretin [51] could also reduce intracellular ROS levels and play an important protective role in oxidative stress damage. As shown in Figure 6, compared to NF-10, purified PCR-C (rich in flavonoids) could effectively reduce *t*-BHP-induced oxidative damage, by increasing SOD activity and GSH level, and reducing ROS and MDA production. Based on the previous studies and present findings, the flavonoids contained in purified PCR-C could be the key components to attenuate *t*-BHP-induced oxidative damage in HepG2 cells. In order to explore whether there was a correlation between antioxidant activities revealed by ROS, MDA, SOD, and GSH contents and the contents of purified total flavonoids, PMFs and individual flavonoids, Pearson’s correlation analysis was performed (Table 5). The content of PMFs was negatively correlated with the ROS levels (R^2^ = −0.939, *p* < 0.01); while the contents of total flavonoids and nobiletin was positively correlated with the SOD activity (R^2^ = 0.928, R^2^ = 0.892, *p* < 0.05). Among the analyzed flavonoids, only 5,6,7,4′-tetramethoxyflavone (R^2^ = −0.970, *p* < 0.01) and nobiletin (R^2^ = −0.953, *p* < 0.05) were negatively correlated with the ROS levels. In addition, 5,6,7,4′-tetramethoxyflavone (R^2^ = 0.879, *p* < 0.05) was also positively correlated with the GSH content. All the results of the correlation analysis demonstrated that 5,6,7,4′-tetramethoxyflavone and nobiletin could be the key components of purified PCR-C to attenuate *t*-BHP-induced oxidative damage in HepG2 cells. A previous study demonstrated that the substitution of the methoxy group in the molecular structure of flavonoids could obviously activate the nuclear factor erythroid 2-related factor 2-antioxidant response element (Nrf2-ARE) antioxidant signal pathway and exert indirect antioxidant activity [52]. Our laboratory also found that tangeretin could activate the mitogen-activated-protein–kinase (MAPK)/Nrf2-ARE antioxidant signal pathway and attenuate oxidative damage [36]. Therefore, with the aging process, the accumulation of PMFs and individual flavonoids in PCR-C might be the key components to the PCR-C antioxidant activity. However, this research model in this study had some limitations. Human hepatocarcinoma HepG2 cell, which by their nature were characterized by significant phenotypic changes of antioxidant defense system. The research results obtained with this model cannot be compared with the normal hepatocyte.

## 4. Conclusions

This study demonstrated, for the first time, that aged PCR-C could attenuate *t*-BHP-induced oxidative damage in HepG2 cells by improving cell viability, increasing SOD activity and GSH content and reducing the overproduction of ROS and MDA. This outcome could be associated with the increase in flavonoids content during aging. In addition, PMFs, mainly 5,6,7,4′-tetramethoxyflavone and nobiletin, were the main functional factors of PCR-C10 to protect HepG2 cells against oxidative damage. This work affirmed the potential role of aged PCR-C as an antioxidant agent in the prevention of oxidative stress and may provide guidance to the classification, authentication, as well as rational use of PCR-C. Further research on the molecular mechanisms for the protective effects of aged PCR-C is recommended.

## Figures and Tables

**Table 1:** Identification of the free flavonoids of PCR-C by HPLC-Q-TOF-MS/MS.

No.	RT (min)	Molecular Formula	[M + H]^+^ (*m*/*z*) (Error, ppm)	Fragment Ions in the Positive Ion Mode	[M-H]^−^ (*m*/*z*) (Error, ppm)	Fragment Ions in the Negative Ion Mode	Identification
1	5.563	C_27_H_30_O_15_	595.1665 (−1.27)	541; 481; 457; 409; 379; 337; 325; 295	593.1520 (−1.34)	503; 473; 455; 383; 353	Vicenin-2
2	5.974	C_28_H_32_O_16_	625.1769 (−1.03)	409; 379; 367; 355; 337; 325; 151	623.1621 (−0.58)	533; 503; 413; 383	Diosmetin-6,8-di-C-glucoside
3	9.022	C_22_H_22_O_11_	463.1238 (−1.26)	445; 427; 367; 343; 313	461.1095 (−1.26)	371; 341; 298	Diosmetin-8-C-glucoside/Scoparin
4	9.278	C_23_H_24_O_12_	493.1345 (−0.92)	329; 314; 299	ND	ND	Coccinoside A
5	10.167	C_28_H_34_O_15_	611.1984 (−0.03)	465; 449; 413; 303	609.1843 (−0.80)	301	Hesperidin
6	25.064	C_20_H_20_O_8_	389.1234 (−0.90)	374; 359; 341	ND	ND	Monohydroxypentamethoxyflavone (1)
7	26.749	C_20_H_20_O_7_	373.1288 (−1.85)	357; 343; 315; 181	ND	ND	Isosinensetin
8	27.688	C_20_H_20_O_8_	389.1238 (−1.90)	374; 359; 355; 323; 305	ND	ND	Monohydroxypentamethoxyflavone (2)
9	31.559	C_20_H_20_O_7_	373.1288 (−1.97)	357; 343; 315; 296; 283; 153	ND	ND	Sinensetin
10	33.255	C_19_H_18_O_6_	343.1182 (−2.12)	313; 285; 181; 153	ND	ND	5,7,8,4′-Tetramethoxyflavone
11	36.562	C_21_H_22_O_8_	403.1405 (−1.35)	373; 355; 327; 211	ND	ND	Nobiletin
12	38.731	C_19_H_18_O_6_	343.1182 (−2.03)	327; 313; 285; 267; 153	ND	ND	5,6,7,4′-Tetramethoxyflavone
13	40.827	C_22_H_24_O_9_	433.1501 (−1.83)	416; 403; 401; 388	ND	ND	3,5,6,7,8,3′,4′-Heptamethoxyflavone
14	44.243	C_20_H_20_O_7_	373.1288 (−2.06)	343; 328; 297; 271; 211	ND	ND	Tangeretin
15	46.599	C_20_H_20_O_8_	389.1238 (−1.85)	373; 359; 341; 313; 197	ND	ND	5-Demethylnobiletin
16	47.436	C_20_H_20_O_9_	405.1554 (0.84)	388; 373; 301; 241	ND	ND	Dihydroxypentamethoxyflavone
17	48.127	C_19_H_18_O_7_	359.1129 (−0.91)	329; 311; 286; 227; 169	ND	ND	Gardenin B

[M + H]^+^, the precursor ion obtained from mass spectrum in positive mode; [M-H]^−^, the precursor ion obtained from mass spectrum in negative mode. RT: retention time, ND: not detected.

**Table 2:** Identification of the bound flavonoids of PCR-C by HPLC-Q-TOF-MS/MS.

No.	RT (Min)	Molecular Formula	[M + H]^+^ (*m*/*z*) (Error, ppm)	Fragment Ions in the Positive Ion Mode	[M-H]^−^ (*m*/*z*) (Error, ppm)	Fragment Ions in the Negative Ion Mode	Identification
1	8.113	C_27_H_30_O_15_	595.1660 (−0.38)	449; 287	593.1504 (0.55)	285	Luteolin 7-O-rutinoside
2	9.005	C_22_H_22_O_11_	463.1232 (0.42)	445; 427; 367; 343; 313; 177	ND	ND	Flavone-C-glucoside
3	9.41	C_27_H_30_O_14_	579.1720 (0.15)	433; 271	577.1553 (−1.03)	269	Isorhoifolin
4	9.744	C_28_H_32_O_15_	609.1821 (−1.11)	301; 286; 177; 153; 71	607.1655 (1.03)	301; 242; 299; 284; 164; 151	Diosmin
5	10.046	C_28_H_32_O_15_	609.1820 (−0.11)	301; 263;153; 85	607.1662 (−0.65)	301; 299; 284; 164	Neodiosmin
6	10.453	C_22_H_24_O_10_	449.1446 (−1.19)	431; 413; 369; 303; 263; 195	ND	ND	Chalcone-C-glycoside
7	10.455	C_28_H_34_O_15_	611.1979 (0.02)	465; 449; 431; 303	609.1827 (−0.14)	301	Hesperidin
8	11.252	C_27_H_32_O_14_	581.1872 (−1.14)	435; 273; 153	579.1719 (0.29)	271; 151	Narirutin
9	12.493	C_28_H_34_O_15_	611.1979 (0.13)	465; 303; 177	609.1827 (−0.16)	301; 286; 242; 164; 151	Neohesperidin
10	16.049	C_28_H_34_O_14_	595.2025 (−0.38)	449; 433; 415; 287; 129; 85	593.1866 (1.08)	285; 270; 164; 151	Didymin
11	19.244	C_28_H_34_O_14_	595.2026 (−0.72)	449; 443; 287; 161	593.1870 (0.97)	327; 309; 285	Poncirin
12	27.484	C_20_H_20_O_7_	373.1284 (0.14)	358; 343; 315	ND	ND	Isosinensetin
13	28.375	C_20_H_20_O_8_	389.1228 (0.65)	374; 359; 341; 313	ND	ND	Monohydroxypentamethoxyflavone
14	32.323	C_20_H_20_O_7_	373.1284 (0.34)	343; 329; 312; 297;151	ND	ND	Sinensetin
15	34.064	C_19_H_18_O_6_	343.1175 (0.18)	328; 313; 285; 153	ND	ND	5,7,8,4′-Tetramethoxyflavone
16	37.922	C_21_H_22_O_8_	403.1394 (−1.49)	388; 373; 355; 327	ND	ND	Nobiletin
17	39.54	C_19_H_18_O_6_	343.1174 (0.56)	327; 313; 309; 282; 153	ND	ND	5,6,7,4′-Tetramethoxyflavone
18	41.631	C_22_H_24_O_9_	433.1490 (0.63)	417; 403; 385; 357	ND	ND	3,5,6,7,8,3′,4′-Heptamethoxyflavone
19	44.723	C_20_H_20_O_7_	373.1288 (−1.52)	343; 325; 297	ND	ND	Tangeretin
20	47.401	C_20_H_20_O_8_	389.1238 (−1.25)	373; 359; 341; 313; 197	ND	ND	5-Demethylnobiletin

[M + H]^+^, the precursor ion obtained from mass spectrum in positive mode; [M-H]^−^, the precursor ion obtained from mass spectrum in negative mode. RT: retention time, ND: not detected.

**Table 3:** Purification fold and total flavonoids content in PCR-C samples after purification.

	Fresh	PCR-C01	PCR-C03	PCR-C05	PCR-C10
Total flavonoids (mg RE/g dw)	102.50 ± 3.94 a	102.50 ± 4.90 a	135.58 ± 5.99 b	139.42 ± 1.61 b	164.04 ± 8.16 c
Purification fold	20.00	10.97	13.81	15.44	11.17

Data were expressed as the mean ± SD (*n* = 3). Means with different letters in each column were significantly different at *p* < 0.05.

**Table 4:** The contents of nine main flavonoids in PCR-C samples after purification.

	Fresh	PCR-C01	PCR-C03	PCR-C05	PCR-C10
Hesperidin	113.64 ± 0.83 ^a^ (59.5)	77.03 ± 1.00 ^b^ (51.1)	87.13 ± 3.58 ^c^ (65.6)	108.61 ± 0.34 ^a^ (67.6)	99.58 ± 6.26 ^d^ (50.2)
Isosinensetin	4.68 ± 0.04 ^a^ (2.5)	3.30 ± 0.06 ^b^ (2.2)	2.09 ± 0.02 ^c^ (1.6)	1.93 ± 0.01 ^d^ (1.2)	4.04 ± 0.02 ^e^ (2.0)
Sinensetin	6.16 ± 0.10 ^a^ (3.2)	4.39 ± 0.05 ^b^ (2.9)	3.22 ± 0.03 ^c^ (2.4)	3.24 ± 0.01 ^c^ (2.0)	6.32 ± 0.02 ^d^ (3.2)
5,7,8,4′-Tetramethoxyflavone	3.08 ± 0.01 ^a^ (1.6)	2.46 ± 0.03 ^b^ (1.6)	1.89 ± 0.01 ^c^ (1.4)	1.85 ± 0.01 ^d^ (1.2)	3.04 ± 0.01 ^a^ (1.5)
Nobiletin	39.97 ± 0.17 ^a^ (20.9)	32.59 ± 0.33 ^b^ (21.6)	23.76 ± 0.13 ^c^ (17.9)	24.44 ± 0.04 ^d^ (15.2)	48.92 ± 0.32 ^e^ (24.7)
5,6,7,4′-Tetramethoxyflavone	0.83 ± 0.05 ^a^ (0.4)	0.82 ± 0.01 ^a^ (0.5)	0.43 ± 0.02 ^b^ (0.3)	0.60 ± 0.01 ^c^ (0.4)	1.30 ± 0.02 ^d^ (0.7)
3,5,6,7,8,3′,4′-Heptamethoxyflavone	3.49 ± 0.03 ^a^ (1.8)	2.71 ± 0.04 ^b^ (1.8)	1.64 ± 0.02 ^c^ (1.2)	1.97 ± 0.01 ^d^ (1.2)	3.57 ± 0.02 ^e^ (1.8)
Tangeretin	18.63 ± 0.09 ^a^ (9.8)	26.80 ± 0.28 ^b^ (17.8)	12.06 ± 0.05 ^c^ (9.1)	17.46 ± 0.06 ^d^ (10.9)	30.68 ± 0.19 ^e^ (15.5)
5-Demethylnobiletin	0.42 ± 0.01 ^a^ (0.2)	0.73 ± 0.01 ^b^ (0.5)	0.60 ± 0.04 ^c^ (0.5)	0.51 ± 0.03 ^d^ (0.3)	0.79 ± 0.00 ^e^ (0.4)
PMFs	77.25 ± 0.36 ^a^ (40.5)	73.80 ± 0.79 ^b^ (48.9)	45.70 ± 0.25 ^c^ (34.4)	51.99 ± 0.12 ^d^ (32.4)	98.66 ± 0.56 ^e^ (49.8)
Total	190.89 ± 1.10 ^a^	150.82 ± 1.78 ^b^	132.83 ± 3.83 ^c^	160.60 ± 0.44 ^d^	198.24 ± 6.04 ^e^

Data were expressed as the mean ± standard deviation (*n* = 3). Means with different letters in each row indicate significant differences among PCR-C of different storage periods (*p* < 0.05). Values in parentheses indicate percentage contribution to the total content (%).

**Table 5:** Pearson correlation coefficient (probability).

Parameter	with ROS Level	with MDA Value	with SOD Activity	with GSH Content
Hesperidin	−0.158	−0.626	0.525	0.147
Isosinensetin	−0.685	−0.380	0.655	0.418
Sinensetin	−0.844	−0.590	0.831	0.640
5,7,8,4′-Tetramethoxyflavone	−0.800	−0.501	0.761	0.569
Nobiletin	**−0.953** *	−0.681	**0.892** *	0.805
5,6,7,4′-Tetramethoxyflavone	**−0.970** **	−0.751	0.871	**0.879** *
3,5,6,7,8,3′,4′-Heptamethoxyflavone	−0.825	−0.593	0.796	0.607
Tangeretin	−0.808	−0.535	0.597	0.718
5-Demethylnobiletin	−0.556	−0.159	0.231	0.619
PMFs	**−0.939** **	−0.646	0.825	0.787
Total flavonoids	−0.813	−0.844	**0.928** *	0.690

* Correlation is significant at the 0.05 level (two-tailed). ** Correlation is significant at the 0.01 level (two-tailed).

## Supplementary Materials

The following are available online at https://www.mdpi.com/article/10.3390/foods11030273/s1, Figure S1: The total ion current (TIC) chromatograms corresponding to positive and negative signals of the free (a and b) and bound (c and d) flavonoids of PCR-C, Figure S2: Chromatogram of nine flavonoids standards at 280 (a) and 330nm (b). (1) Hesperidin, (2) Isosinensetin, (3) Sinensetin, (4) 5,7,8,4’-Tetramethoxyflavone, (5) Nobiletin, (6) 5,6,7,4’-Tetramethoxyflavone, (7) 3,5,6,7,8,3’,4’-Heptamethoxyflavone, (8) Tangeretin, (9) 5-Demethylnobiletin, Table S1: Calibration curve data for nine reference standards by HPLC, Table S2: Exact values of p between nine free, bound and total flavonoids in PCR-C10 and other samples (by Duncan’s test and Tamhane’s test).
