# Supplementary material for: Aged Pericarpium Citri Reticulatae ‘Chachi’ Attenuates Oxidative Damage Induced by tert-Butyl Hydroperoxide (t-BHP) in HepG2 Cells
Source: Foods. 2022 Jan 20;11(3):273. doi: 10.3390/foods11030273 (PMC8834029; doi:10.3390/foods11030273)
Supplement: Supplementary file 1 [file foods-11-00273-s001.zip › foods-1454772-supplementary.pdf]

Supplementary Materials

Table S1: Calibration curve data for nine reference standards by HPLC

	λ(nm)	RT (min)	Concentration range (mg/L)	Regression equation [a]	R^2	LOQ [b] (mg/L)	LOD [b] (mg/L)	Precision (RSD %, n=6)		Repeatability (RSD %, n=6)		Recovery (%, n=6)		Recovery (RSD %, n=6)	
								Intra	Inter	Free	Bound	Free	Bound	Free	Bound
Hesperidin	280	9.71	4.77-610.56	Y = 29239x - 94574	1	2.55	0.77	0.15	1.72	2.83	0.96	99.13	101.49	0.93	1.75
Isosinensetin	330	26.19	0.29-37.24	Y = 31002x - 21553	0.9999	0.16	0.05	1.25	1.00	0.75	4.06	99.13	99.01	0.93	1.79
Sinensetin	330	30.95	0.29-37.24	Y = 39319x - 25690	0.9999	0.16	0.05	0.36	0.53	0.58	3.07	100.10	100.99	0.92	1.75
5,7,8,4'-Tetramethoxyflavone	330	32.71	0.27-34.23	Y = 37986x - 23186	0.9999	0.14	0.04	1.42	1.76	1.24	3.69	101.00	99.33	0.91	1.78
Nobiletin	330	36.45	1.89-241.43	Y = 70725x - 154751	0.9999	1.01	0.30	0.17	1.92	0.46	1.29	100.35	102.50	0.92	1.73
5,6,7,4'-Tetramethoxyflavone	330	38.12	0.27-34.23	Y = 76182x - 37481	0.9999	0.14	0.04	0.69	0.99	0.97	4.42	99.71	101.51	0.93	1.74
3,5,6,7,8,3',4'-Heptamethoxyflavone	330	40.12	0.34-43.24	Y = 49410x - 28659	0.9999	0.18	0.05	0.55	1.68	0.63	1.65	100.01	97.40	0.92	1.82
Tangeretin	330	43.70	1.75-223.42	Y = 51739x - 112431	0.9999	0.93	0.28	0.11	1.60	0.50	0.81	100.01	98.61	0.92	1.80
5-Demethylnobiletin	330	46.22	0.30-38.84	Y= 51751x - 25413	0.9998	0.16	0.05	0.09	1.23	0.43	3.12	102.08	98.17	0.90	1.80

[a] Where x and Y denote the corresponding injection concentration (μmol/L) and peak area of the samples respectively. [b] The LOQ and LOD were

determined by SNR (signal-to-noise ratio) method.

Table S2: Exact values of p between nine free, bound and total flavonoids in PCR-C10 and other samples (by Duncan's test and Tamhane's test)

	Fresh			PCR-C01			PCR-C03			PCR-C05		
	Free	Bound	Total	Free	Bound	Total	Free	Bound	Total	Free	Bound	Total
Hesperidin	0.026	0.000	0.562	0.007	0.017	0.018	0.031	0.002	0.002	0.088	0.000	0.001
Isosinensetin	0.000	nd	0.034	0.000	0.005	0.013	0.000	0.002	0.000	0.000	nd	0.009
Sinensetin	0.000	0.001	0.034	0.000	0.005	0.008	0.000	0.002	0.001	0.000	0.001	0.015
5,7,8,4′-Tetramethoxyflavone	0.000	nd	0.012	0.000	0.002	0.000	0.000	0.002	0.000	0.000	0.001	0.000
Nobiletin	0.000	0.000	0.000	0.000	0.000	0.000	0.000	0.001	0.000	0.000	0.000	0.000
5,6,7,4′-Tetramethoxyflavone	0.000	nd	0.000	0.000	0.026	0.000	0.000	0.026	0.000	0.000	nd	0.000
3,5,6,7,8,3′,4′-Heptamethoxyflavone	0.002	nd	0.038	0.000	0.001	0.012	0.000	0.008	0.002	0.000	nd	0.013
Tangeretin	0.000	0.000	0.000	0.000	0.000	0.000	0.000	0.000	0.000	0.000	0.000	0.000
5-Demethylnobiletin	0.000	0.002	0.031	0.000	0.005	0.030	0.000	0.017	0.021	0.000	0.001	0.019
PMFs	0.000	0.000	0.000	0.000	0.000	0.000	0.000	0.000	0.000	0.000	0.000	0.000

nd, not detected in the samples, so the significance analysis was not available.

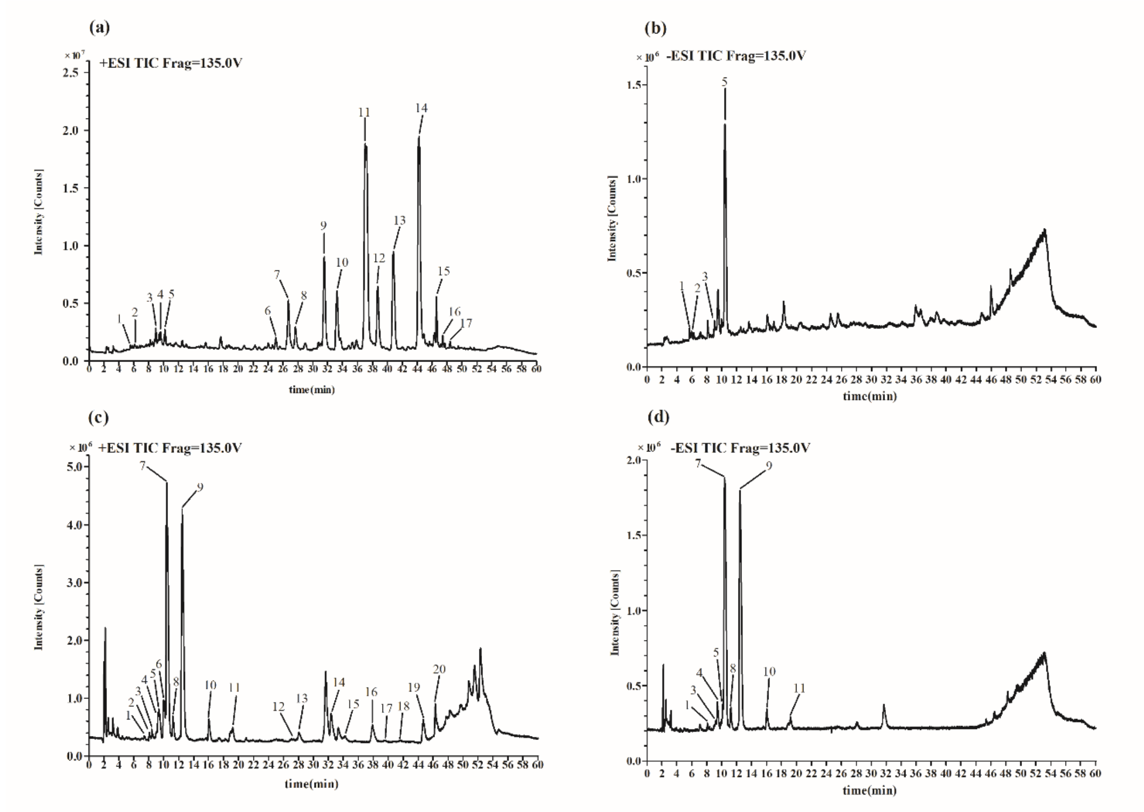

Figure S1. The total ion current (TIC) chromatograms corresponding to positive and negative signals of the free (a and b) and bound (c and d) flavonoids of PCR-C

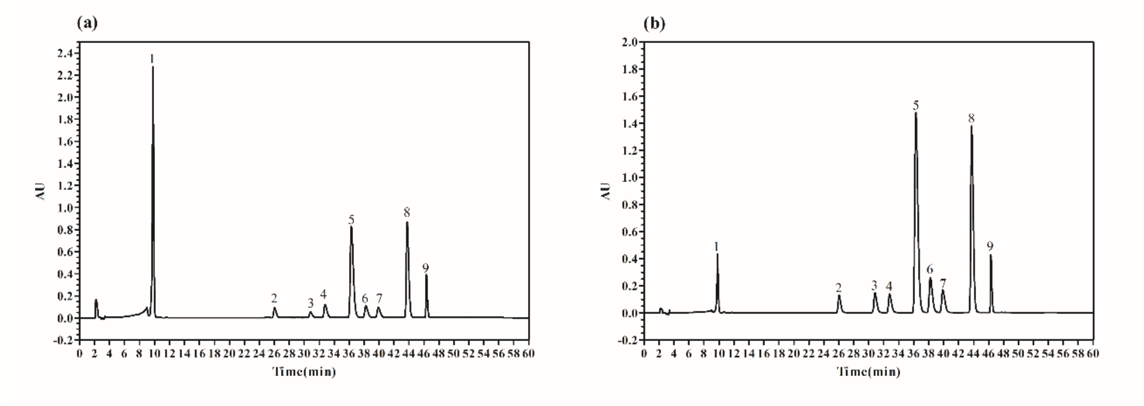

Figure S2. Chromatogram of nine flavonoids standards at 280 (a) and 330nm (b). (1) Hesperidin, (2) Isosinensetin, (3) Sinensetin, (4) 5,7,8,4'-Tetramethoxyflavone, (5) Nobiletin, (6) 5,6,7,4'-Tetramethoxyflavone, (7) 3,5,6,7,8,3',4'-Heptamethoxyflavone, (8) Tangeretin, (9) 5-Demethylnobiletin
